# 2-(2-Meth­oxy­phen­yl)-1-benzofuran

**DOI:** 10.1107/S1600536811017168

**Published:** 2011-05-14

**Authors:** Michaela Pojarová, Michal Dušek, Andrej Jančařík, Emanuel Makrlík, Zdeňka Sedláková

**Affiliations:** a Institute of Physics, AS CR, v.v.i., Na Slovance 2, 182 21 Prague 8, Czech Republic; b Institute of Organic Chemistry and Biochemistry of AS CR, Fleming sq. 2, 166 10 Prague 6, Czech Republic; c Faculty of Applied Sciences, University of West Bohemia, Husova 11, 306 14 Pilsen, Czech Republic; d Institue od Macromolecular Chemistry of AS CR, Heyrovský sq. 2, 162 06 Prague 6, Czech Republic

## Abstract

In the title compound, C_15_H_12_O_2_, the dihedral angle between the aromatic ring systems is 16.67 (6)°. The methyl C atom is almost coplanar with its attached benzene ring [displacement = 0.020 (2) Å]. In the crystal, the mol­ecules are connected by weak C—H⋯O bonds and face-to-edge C—H⋯π inter­actions between the 2-meth­oxy­phenyl rings.

## Related literature

For the biological activity of related compounds, see: Akgul & Anil (2003 ▶); Aslam *et al.* (2006 ▶); Galal *et al.* (2009 ▶); Khan *et al.* (2005 ▶); Soekamto *et al.* (2003 ▶). For the synthesis, see: Takeda *et al.* (2007 ▶).
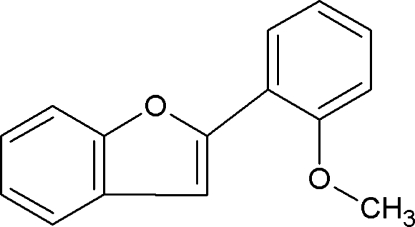


## Experimental

### 

#### Crystal data

C_15_H_12_O_2_
*M* *_r_* = 224.25
Orthorhombic, 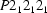

*a* = 6.9419 (1) Å
*b* = 11.4409 (2) Å
*c* = 14.1703 (3) Å
*V* = 1125.43 (3) Å^3^
*Z* = 4
Cu *K*α radiation
μ = 0.70 mm^−1^
*T* = 120 K
0.27 × 0.25 × 0.12 mm

#### Data collection

Oxford Diffraction Xcalibur Atlas Gemini Ultra diffractometer
Absorption correction: multi-scan (*CrysAlis PRO*; Oxford Diffraction, 2010 ▶) *T*_min_ = 0.683, *T*_max_ = 1.000
11347 measured reflections
2006 independent reflections
1955 reflections with *I* > 2σ(*I*)
*R*_int_ = 0.050

#### Refinement

*R*[*F* ^2^ > 2σ(*F* ^2^)] = 0.033
*wR*(*F* ^2^) = 0.088
*S* = 1.09
2006 reflections
155 parameters
H-atom parameters constrained
Δρ_max_ = 0.20 e Å^−3^
Δρ_min_ = −0.14 e Å^−3^
Absolute structure: Flack (1983 ▶), 822 Friedel pairs
Flack parameter: −0.2 (2)

### 

Data collection: *CrysAlis PRO* (Oxford Diffraction, 2010 ▶); cell refinement: *CrysAlis PRO*; data reduction: *CrysAlis PRO*; program(s) used to solve structure: *SHELXS97* (Sheldrick, 2008 ▶); program(s) used to refine structure: *SHELXL97* (Sheldrick, 2008 ▶); molecular graphics: *Mercury* (Macrae *et al.*, 2006 ▶); software used to prepare material for publication: *publCIF* (Westrip, 2010 ▶).

## Supplementary Material

Crystal structure: contains datablocks I, global. DOI: 10.1107/S1600536811017168/hb5866sup1.cif

Structure factors: contains datablocks I. DOI: 10.1107/S1600536811017168/hb5866Isup2.hkl

Additional supplementary materials:  crystallographic information; 3D view; checkCIF report

## Figures and Tables

**Table 1:** Hydrogen-bond geometry (Å, °) *Cg*1 is the centroid of the C2–C7 ring.

*D*—H⋯*A*	*D*—H	H⋯*A*	*D*⋯*A*	*D*—H⋯*A*
C1—H1*B*⋯O2^i^	0.96	2.57	3.272 (2)	131
C3—H3⋯*Cg*1^ii^	0.93	2.78	3.604 (2)	149

Symmetry codes: (i) 
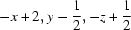
; (ii) 
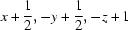
.

**Fig. 1.**
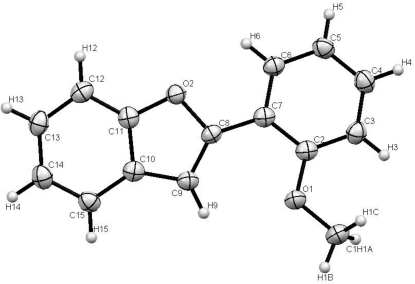
View of (I) with displacement ellipsoids shown at the 50% probability level.

**Fig. 2.**
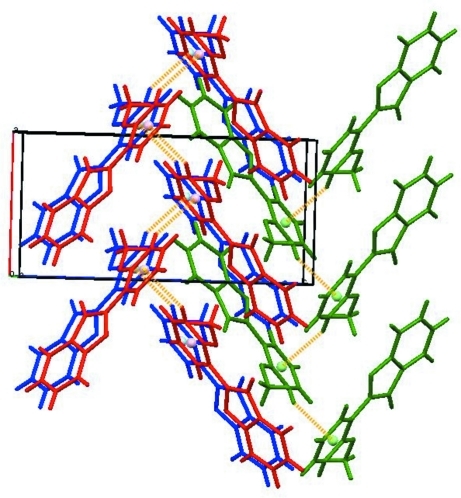
Projection along the b axis with highlighted face-to-edge CH-π interactions between methoxyphenyl rings.

## supplementary crystallographic information

### Comment

A wide range of natural products with diverse pharmaceutical properties, such as antifungal, antitumor, antiviral, and antimicrobial (Aslam *et al.*, 2006; Galal *et al.*, 2009; Khan *et al.*, 2005), contain a benzofuran ring (Akgul & Anil, 2003; Soekamto *et al.*, 2003). In this paper, we present a crystal structure of the title compound, (I).

The benzofuran unit is essentially planar, with a mean deviation of 0.019 (2)Å from the least-square plane defined by the nine atoms in benzofuran ring. The methoxy group forms intermolecular hydrogen bond to the oxygen in benzofuran ring (Table 1). Another weak interactions found in the crystal is the C—H···π interaction between the 2-methoxyphenyl rings [C3—H3···Cg1 (C2 → C7)] which is responsible for their edge-to-face orientation (Fig. 2).

### Experimental

2-(2'-methoxyphenyl]-benzo[*b*]furan was synthesized by the method described by Takeda (Takeda *et al.*, 2007). Crystals were prepared by slow evaporation from acetonitrile.

### Refinement

The hydrogen atoms were localized from the difference Fourier map. Despite of that, all hydrogen atoms connected to C were constrained to ideal positions. The isotropic temperature parameters of hydrogen atoms were calculated as 1.2**U*_eq_ of the parent atom.

### Crystal data

**Table:**

C_15_H_12_O_2_	*F*(000) = 472
*M**_r_* = 224.25	*D*_x_ = 1.323 Mg m^−^^3^
Orthorhombic, *P*2_1_2_1_2_1_	Cu *K*α radiation, λ = 1.5418 Å
Hall symbol: P 2ab 2ac	Cell parameters from 7843 reflections
*a* = 6.9419 (1) Å	θ = 3.1–66.9°
*b* = 11.4409 (2) Å	µ = 0.70 mm^−^^1^
*c* = 14.1703 (3) Å	*T* = 120 K
*V* = 1125.43 (3) Å^3^	Plate, colourless
*Z* = 4	0.27 × 0.25 × 0.12 mm

### Data collection

**Table:**

Oxford Diffraction Xcalibur Atlas Gemini ultra diffractometer	2006 independent reflections
Radiation source: Enhance Ultra (Cu) X-ray Source	1955 reflections with *I* > 2σ(*I*)
mirror	*R*_int_ = 0.050
Detector resolution: 10.3748 pixels mm^-1^	θ_max_ = 67.1°, θ_min_ = 5.0°
Rotation method data acquisition using ω scans	*h* = −8→7
Absorption correction: multi-scan (*CrysAlis PRO*; Oxford Diffraction, 2010)	*k* = −13→13
*T*_min_ = 0.683, *T*_max_ = 1.000	*l* = −16→14
11347 measured reflections	

### Refinement

**Table:**

Refinement on *F*^2^	Secondary atom site location: difference Fourier map
Least-squares matrix: full	Hydrogen site location: inferred from neighbouring sites
*R*[*F*^2^ > 2σ(*F*^2^)] = 0.033	H-atom parameters constrained
*wR*(*F*^2^) = 0.088	*w* = 1/[σ^2^(*F*_o_^2^) + (0.0543*P*)^2^ + 0.153*P*] where *P* = (*F*_o_^2^ + 2*F*_c_^2^)/3
*S* = 1.09	(Δ/σ)_max_ < 0.001
2006 reflections	Δρ_max_ = 0.20 e Å^−^^3^
155 parameters	Δρ_min_ = −0.14 e Å^−^^3^
0 restraints	Absolute structure: Flack (1983), 822 Friedel pairs
Primary atom site location: structure-invariant direct methods	Flack parameter: −0.2 (2)

### Special details

**Table:**

Geometry. All e.s.d.'s (except the e.s.d. in the dihedral angle between two l.s. planes) are estimated using the full covariance matrix. The cell e.s.d.'s are taken into account individually in the estimation of e.s.d.'s in distances, angles and torsion angles; correlations between e.s.d.'s in cell parameters are only used when they are defined by crystal symmetry. An approximate (isotropic) treatment of cell e.s.d.'s is used for estimating e.s.d.'s involving l.s. planes.
Refinement. Refinement of *F*^2^ against ALL reflections. The weighted *R*-factor *wR* and goodness of fit *S* are based on *F*^2^, conventional *R*-factors *R* are based on *F*, with *F* set to zero for negative *F*^2^. The threshold expression of *F*^2^ > σ(*F*^2^) is used only for calculating *R*-factors(gt) *etc*. and is not relevant to the choice of reflections for refinement. *R*-factors based on *F*^2^ are statistically about twice as large as those based on *F*, and *R*- factors based on ALL data will be even larger. The hydrogen atoms were localized from the difference Fourier map. Despite of that,all hydrogen atoms connected to C were constrained to ideal positions. The isotropic temperature parameters of hydrogen atoms were calculated as 1.2**U*_eq_ of the parent atom.

### Fractional atomic coordinates and isotropic or equivalent isotropic displacement parameters (Å^2^)

**Table:**

	*x*	*y*	*z*	*U*_iso_*/*U*_eq_	
O1	1.06771 (15)	0.13587 (9)	0.33592 (7)	0.0323 (3)	
O2	0.61359 (14)	0.36286 (9)	0.28934 (7)	0.0293 (3)	
C2	1.0779 (2)	0.24444 (13)	0.37522 (10)	0.0282 (3)	
C11	0.4903 (2)	0.32298 (13)	0.22083 (10)	0.0281 (3)	
C6	0.9320 (2)	0.43475 (13)	0.38967 (10)	0.0301 (3)	
H6	0.8355	0.4875	0.3737	0.036*	
C7	0.9277 (2)	0.32219 (13)	0.35139 (10)	0.0275 (3)	
C3	1.2221 (2)	0.27897 (14)	0.43667 (11)	0.0319 (3)	
H3	1.3202	0.2272	0.4525	0.038*	
C15	0.4610 (2)	0.17104 (13)	0.10446 (11)	0.0322 (3)	
H15	0.5069	0.1044	0.0743	0.039*	
C9	0.7486 (2)	0.20323 (12)	0.22207 (11)	0.0295 (3)	
H9	0.8343	0.1430	0.2087	0.035*	
C14	0.2874 (2)	0.22039 (14)	0.07865 (11)	0.0344 (4)	
H14	0.2165	0.1869	0.0300	0.041*	
C5	1.0769 (2)	0.46960 (13)	0.45085 (11)	0.0333 (3)	
H5	1.0774	0.5449	0.4755	0.040*	
C10	0.5664 (2)	0.22374 (13)	0.17718 (10)	0.0283 (3)	
C8	0.7723 (2)	0.28854 (12)	0.28798 (10)	0.0268 (3)	
C13	0.2159 (2)	0.31977 (14)	0.12430 (11)	0.0346 (4)	
H13	0.0980	0.3505	0.1055	0.041*	
C12	0.3160 (2)	0.37352 (14)	0.19675 (11)	0.0333 (3)	
H12	0.2690	0.4395	0.2274	0.040*	
C1	1.2147 (3)	0.05403 (14)	0.36035 (12)	0.0382 (4)	
H1A	1.3373	0.0828	0.3394	0.046*	
H1B	1.1884	−0.0196	0.3305	0.046*	
H1C	1.2171	0.0438	0.4276	0.046*	
C4	1.2206 (2)	0.39125 (14)	0.47490 (11)	0.0352 (4)	
H4	1.3168	0.4136	0.5168	0.042*	

### Atomic displacement parameters (Å^2^)

**Table:**

	*U*^11^	*U*^22^	*U*^33^	*U*^12^	*U*^13^	*U*^23^
O1	0.0364 (5)	0.0258 (5)	0.0348 (5)	0.0044 (5)	−0.0035 (5)	−0.0030 (4)
O2	0.0288 (5)	0.0280 (5)	0.0311 (5)	0.0011 (4)	0.0007 (4)	−0.0013 (4)
C2	0.0321 (7)	0.0253 (7)	0.0272 (7)	−0.0002 (6)	0.0043 (6)	0.0028 (5)
C11	0.0276 (6)	0.0282 (7)	0.0284 (7)	−0.0049 (6)	0.0025 (6)	0.0041 (6)
C6	0.0328 (8)	0.0259 (7)	0.0317 (7)	0.0009 (6)	0.0005 (6)	0.0025 (6)
C7	0.0300 (7)	0.0253 (7)	0.0271 (7)	−0.0013 (6)	0.0031 (6)	0.0029 (6)
C3	0.0322 (7)	0.0303 (7)	0.0331 (8)	0.0005 (7)	−0.0021 (6)	0.0049 (6)
C15	0.0370 (8)	0.0273 (7)	0.0324 (7)	−0.0031 (6)	−0.0008 (6)	0.0006 (6)
C9	0.0320 (7)	0.0251 (7)	0.0314 (8)	0.0011 (6)	0.0007 (6)	−0.0008 (6)
C14	0.0351 (8)	0.0332 (8)	0.0349 (8)	−0.0076 (7)	−0.0039 (7)	0.0039 (6)
C5	0.0388 (8)	0.0257 (7)	0.0352 (8)	−0.0034 (7)	−0.0023 (7)	−0.0010 (6)
C10	0.0307 (7)	0.0251 (7)	0.0291 (7)	−0.0032 (6)	0.0026 (6)	0.0033 (6)
C8	0.0279 (6)	0.0235 (7)	0.0289 (7)	0.0008 (6)	0.0030 (6)	0.0033 (6)
C13	0.0276 (7)	0.0372 (8)	0.0389 (8)	−0.0024 (7)	−0.0013 (6)	0.0072 (7)
C12	0.0318 (7)	0.0309 (8)	0.0373 (8)	0.0014 (7)	0.0046 (6)	0.0029 (6)
C1	0.0394 (8)	0.0316 (8)	0.0437 (9)	0.0092 (7)	−0.0027 (7)	−0.0036 (7)
C4	0.0382 (8)	0.0326 (8)	0.0348 (8)	−0.0048 (7)	−0.0059 (7)	0.0015 (6)

### Geometric parameters (Å, °)

**Table:**

O1—C2	1.3630 (18)	C15—H15	0.9300
O1—C1	1.4273 (18)	C9—C8	1.361 (2)
O2—C11	1.3723 (18)	C9—C10	1.435 (2)
O2—C8	1.3921 (17)	C9—H9	0.9300
C2—C3	1.384 (2)	C14—C13	1.399 (2)
C2—C7	1.411 (2)	C14—H14	0.9300
C11—C12	1.384 (2)	C5—C4	1.384 (2)
C11—C10	1.397 (2)	C5—H5	0.9300
C6—C5	1.386 (2)	C13—C12	1.384 (2)
C6—C7	1.398 (2)	C13—H13	0.9300
C6—H6	0.9300	C12—H12	0.9300
C7—C8	1.456 (2)	C1—H1A	0.9600
C3—C4	1.394 (2)	C1—H1B	0.9600
C3—H3	0.9300	C1—H1C	0.9600
C15—C14	1.380 (2)	C4—H4	0.9300
C15—C10	1.400 (2)		
			
C2—O1—C1	117.50 (12)	C13—C14—H14	119.3
C11—O2—C8	106.31 (11)	C4—C5—C6	119.38 (14)
O1—C2—C3	123.68 (14)	C4—C5—H5	120.3
O1—C2—C7	116.00 (14)	C6—C5—H5	120.3
C3—C2—C7	120.31 (13)	C11—C10—C15	118.58 (14)
O2—C11—C12	125.51 (14)	C11—C10—C9	105.67 (13)
O2—C11—C10	110.34 (13)	C15—C10—C9	135.74 (15)
C12—C11—C10	124.14 (14)	C9—C8—O2	110.60 (12)
C5—C6—C7	121.56 (14)	C9—C8—C7	134.67 (14)
C5—C6—H6	119.2	O2—C8—C7	114.61 (11)
C7—C6—H6	119.2	C12—C13—C14	121.74 (15)
C6—C7—C2	118.17 (14)	C12—C13—H13	119.1
C6—C7—C8	119.94 (13)	C14—C13—H13	119.1
C2—C7—C8	121.89 (13)	C11—C12—C13	115.87 (15)
C2—C3—C4	120.13 (15)	C11—C12—H12	122.1
C2—C3—H3	119.9	C13—C12—H12	122.1
C4—C3—H3	119.9	O1—C1—H1A	109.5
C14—C15—C10	118.36 (15)	O1—C1—H1B	109.5
C14—C15—H15	120.8	H1A—C1—H1B	109.5
C10—C15—H15	120.8	O1—C1—H1C	109.5
C8—C9—C10	107.07 (13)	H1A—C1—H1C	109.5
C8—C9—H9	126.5	H1B—C1—H1C	109.5
C10—C9—H9	126.5	C5—C4—C3	120.42 (15)
C15—C14—C13	121.31 (15)	C5—C4—H4	119.8
C15—C14—H14	119.3	C3—C4—H4	119.8

### Hydrogen-bond geometry (°)

**Table:**

Cg1 is the centroid of the C2–C7 ring.

**Table:**

*D*—H···*A*
—···
—···
